# Blunting Neuroinflammation by Targeting the Immunoproteasome with Novel Amide Derivatives

**DOI:** 10.3390/ijms241310732

**Published:** 2023-06-27

**Authors:** Chiara Imbesi, Roberta Ettari, Natasha Irrera, Maria Zappalà, Giovanni Pallio, Alessandra Bitto, Federica Mannino

**Affiliations:** 1 Department of Clinical and Experimental Medicine, University of Messina, Via C. Valeria, 98125 Messina, Italy; chimbesi@unime.it (C.I.); nirrera@unime.it (N.I.); gpallio@unime.it (G.P.); fmannino@unime.it (F.M.); 2 Department of Chemical, Biological, Pharmaceutical and Environmental Sciences, University of Messina, Viale Ferdinando Stagno D’Alcontres, 98166 Messina, Italy; roberta.ettari@unime.it (R.E.); mzappala@unime.it (M.Z.)

**Keywords:** amide derivates, neuroinflammation, immunoproteasome, oxidative stress

## Abstract

Neuroinflammation is an inflammatory response of the nervous tissue mediated by the production of cytokines, chemokines, and reactive oxygen species. Recent studies have shown that an upregulation of immunoproteasome is highly associated with various diseases and its inhibition attenuates neuroinflammation. In this context, the development of non-covalent immunoproteasome-selective inhibitors could represent a promising strategy for treating inflammatory diseases. Novel amide derivatives, KJ3 and KJ9, inhibit the β5 subunit of immunoproteasome and were used to evaluate their possible anti-inflammatory effects in an in vitro model of TNF-α induced neuroinflammation. Differentiated SH-SY5Y and microglial cells were challenged with 10 ng/mL TNF-α for 24 h and treated with KJ3 (1 µM) and KJ9 (1 µM) for 24 h. The amide derivatives showed a significant reduction of oxidative stress and the inflammatory cascade triggered by TNF-α reducing p-ERK expression in treated cells. Moreover, the key action of these compounds on the immunoproteasome was further confirmed by halting the IkB-α phosphorylation and the consequent inhibition of NF-kB. As downstream targets, IL-1β and IL-6 expression resulted also blunted by either KJ3 and KJ9. These preliminary results suggest that the effects of these two compounds during neuroinflammatory response relies on the reduced expression of pro-inflammatory targets.

## 1. Introduction

Neuroinflammation is an inflammatory response of the nervous tissue mediated by the production of cytokines, chemokines, and reactive oxygen species. Recent studies have shown that an upregulation of immunoproteasome is highly associated with various diseases and its inhibition attenuates neuroinflammation. The immunoproteasome (iP) is a specialized, pro-inflammatory cytokine-, and oxidative stress-induced subtype of the constitutive proteasome and plays a key role in both immuno and non-immuno responses due to its three immunosubunits. In detail, in presence of inflammatory stimuli, such as IFN-γ and TNF-α, or oxidative stress, the proteolytic β-subunits of 20S core constitutive proteasome, i.e., β1c, β2c, and β5c, are substituted by immunosubunits β1i (LMP2/proteasome subunit beta 9, PSMB9), β2i (multicatalytic endopeptidase complex-like 1, MECL-1) and β5i (LMP7). As a consequence of this subunit replacement, the iP characterized by enhanced proteolytic activities compared to the constitutive proteasome was formed [1,2].

The iP is mainly involved in the MHC-I antigen process, mediating the production of inflammatory cytokines in several animal model [3,4], and regulating protein homeostasis preventing the accumulation of the oxidatively-damaged and ubiquitylated proteins [5]. iP is also involved in a wide number of inflammatory diseases, such as inflammatory bowel disease, autoimmune disease, rheumatoid arthritis, systemic lupus erythematosus, and neuroinflammation [6]. In particular, different works demonstrated that iP is expressed in neurons, glia, astrocytes, and endothelial cells of the central nervous system (CNS), and it is markedly upregulated by pro-inflammatory stimuli, such as LPS, IFN-γ, and TNF-α [7,8,9,10]. In particular, under inflammatory or stress conditions of the nervous system, the accumulation of misfolded proteins causes the production of danger-associated molecular pattern molecules (DAMPs) which stimulate NF-kB activation and the upregulation of iP binding Toll-like receptor 9 (TLR-9) [11,12]. 

**Figure 1:**
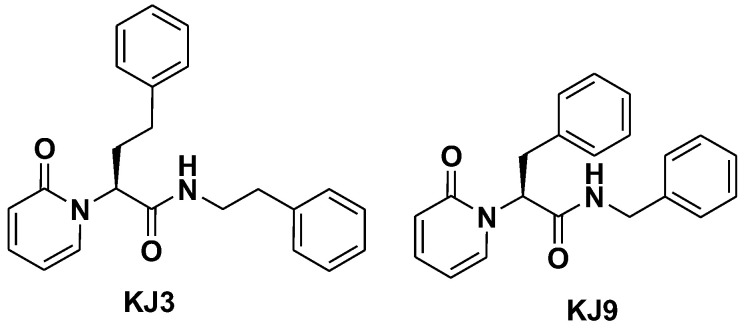
Structure of amide derivatives KJ3 and KJ9.

In this context, neuroinflammation would trigger proteotoxicity and the persistent upregulation of iP, which in turn should be a key promoter of neuroinflammation [13]. In this context, immunoproteasome inhibition could represent a hopeful strategy for the management of several human diseases including inflammatory disease. In recent years, several β5i and/or β1i immunoproteasome-selective inhibitors have been developed; in particular, Ettari et al. [14]. identified several amide derivatives, which are able to inhibit the chymotrypsin-like activities of iP and, due to the non-covalent inhibition, reduce side effects associated with the irreversible inhibition. Among these, KJ3 and KJ9 (Figure 1) are characterized by the presence of a pyridone scaffold at the P3 site which acts as bioisostere of a leucine residue, at the P2 site they bear a homophenyl alanine (KJ3) or a phenyl alanine residue (KJ9), while the amide function spanning into the P1 region is *N*-phenyl ethyl (KJ3) or *N*-benzyl substituted (KJ9). 

KJ9 and KJ3 were already examined for their capability to inhibit each catalytic subunit of c20S and i20S. The results of this investigation showed that KJ9 inhibited both the β5c and the β5i subunits with *K*_i_ values of 3.02 and 7.77 µM, while KJ3 inhibited the sole β5i subunit with a *K*_i_ value of 3.85 µM [14]. In particular, unlike the covalent inhibitors, these designed analogues don’t own an electrophilic moiety and could act as noncovalent immunoproteasome inhibitors also reducing the side effects due to the irreversible inhibition [15]. Therefore, the goal of this work was to investigate the effects of amide derivatives, KJ3 and KJ9, in an in vitro model TNF-α induced neuroinflammation in differentiated SH-SY5Y and HMC3 cells.

## 2. Results 

### 2.1. Proteasome and Immunoproteasome Inhibition Assays

The synthesis of amides KJ3 and KJ9 was achieved in agreement with our previously reported procedure [14]. 

Herein, in the present study, we tested the two amides against the constitutive and immuno-subunits, on which they were proven to be active, to calculate the relative IC_50_ values, and based on these data to further evaluate KJ3 and KJ9 at the cellular level. 

We measured the rate of hydrolysis of the appropriate fluorogenic substrate, i.e., Suc-Leu-Leu-Val-Tyr-AMC for both β5i and β5c. MG-132 (Z-Leu-Leu-Leu-al), a reversible inhibitor of both proteasome and immunoproteasome, was used as a positive control. 

Tian continuous assays were conducted using seven different concentrations, ranging from those that minimally inhibited to those that fully inhibited the immunoproteasome or the proteasome subunits to determine the IC_50_ values. 

Between the two tested compounds, amide KJ9 inhibited both the chymotrypsin-like activities of proteasome and immunoproteasome with an IC_50_ of 6.90 ± 0.18 µM for β5c and 16.52 ± 0.35 for β5i, respectively. On the contrary, KJ3 inhibited the sole β5i of immunoproteasome with an IC_50_ value of 7.61 ± 0.76. 

### 2.2. Cytotoxicity Effects of Amide Derivates, KJ3 and KJ9, in Neurons and Microglia Cells

**Figure 2:**
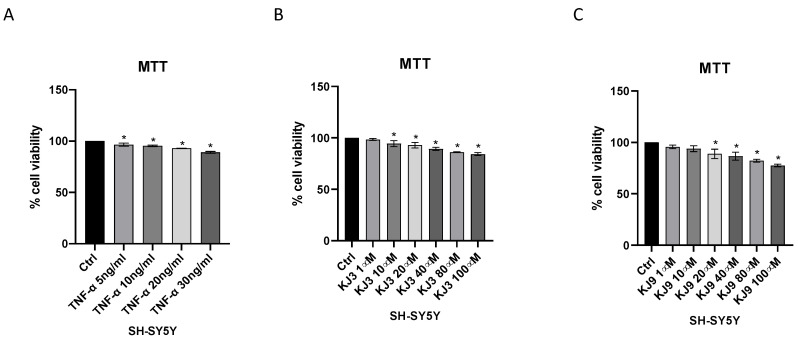
Cytotoxicity assay evaluated by MTT in the differentiated SH-SY5Y cell line stimulated with TNF-α (10 ng/mL) (**A**) for 24 h and then treated with KJ3 (1 μM) (**B**) or KJ9 (1 μM) (**C**) for 24 h. The data are expressed as means ± SD; * *p* < 0.05 vs. Ctrl.

**Figure 3:**
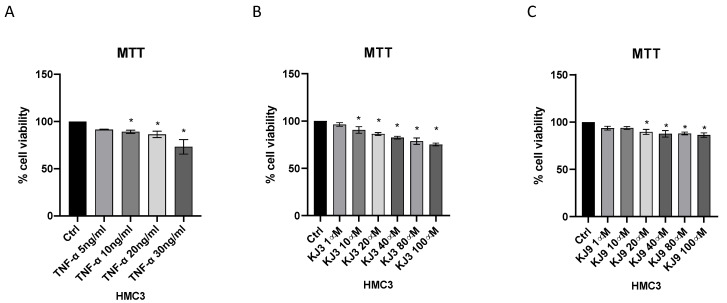
Cytotoxicity assay evaluated by MTT in HMC3 cell line stimulated with TNF-α (10 ng/mL) (**A**) for 24 h and then treated with KJ3 (1 μM) (**B**) or KJ9 (1 μM) (**C**) for 24 h. The data are expressed as means ± SDs; * *p* < 0.05 vs. Ctrl.

Cytotoxic effects of KJ3 and KJ9 were evaluated in differentiated SH-SY5Y and HMC3 cells using MTT assay. In particular, cells were plated in a 96-well plate at a density of 1 × 10^5^ cells/well and treated with TNF-α, KJ3, or KJ9 for 24 h. TNF-α stimulation reduced cell viability at all tested doses in SH-SY5Y cells (Figure 2A), instead in the HMC3 cell line, the challenge with TNF-α caused a reduction of viability starting from the dose of 10 ng/mL (Figure 3A). KJ3 affected cell viability starting from 10 µM while KJ9 started from 20 µM in differentiated SH-SY5Y (Figure 2B,C) and in the HMC3 cell line (Figure 3B,C).

### 2.3. KJ3 and KJ9 Decrease the Inflammatory Targets

**Figure 4:**
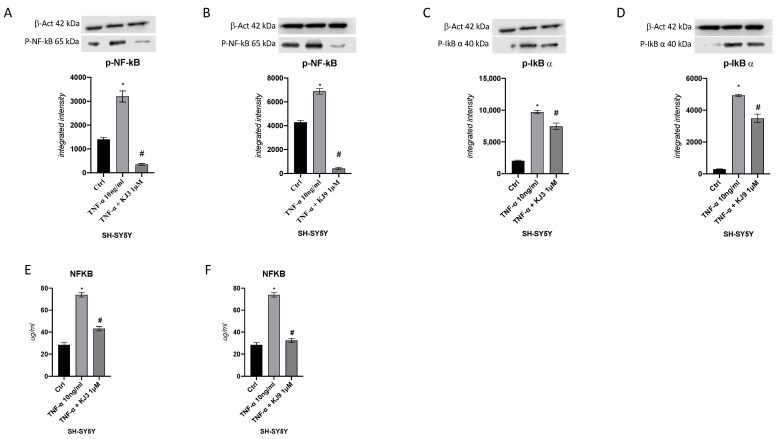
The graphs show NFkb protein level evaluated by ELISA assay (**E**,**F**) and Western blot (**A**,**B**) and p-IkBα protein expression evaluated by Western blot (**C**,**D**) in differentiated SH-SY5Y cells treated with KJ3 (1 μM) or KJ9 (1 μM) for 24 h following TNF-α (10 ng/mL) stimulation. The data are expressed as means ± SDs. * *p* < 0.05 vs. CTRL; # *p* < 0.05 vs. TNF-α.

**Figure 5:**
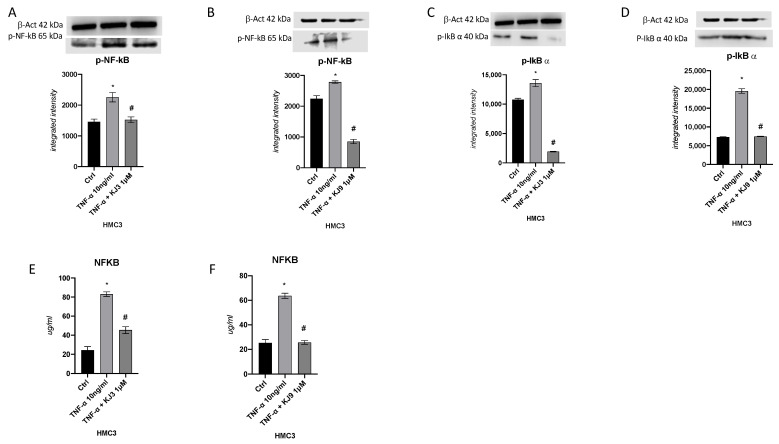
The graphs show NFkb protein level evaluated by ELISA assay (**E**,**F**) and Western blot (**A**,**B**) and p-IkBα protein expression evaluated by Western blot (**C**,**D**) in HMC3 cell line treated with KJ3 (1 μM) or KJ9 (1 μM) for 24 h following TNF-α (10 ng/mL) stimulation. The data are expressed as means ± SDs. * *p* < 0.05 vs. CTRL; # *p* < 0.05 vs. TNF-α.

The protein level of the p-NF-kb and p-IkB-α were investigated to determine if the two tested compounds were able to reduce TNF-α induced neuroinflammation in both cell lines. In particular, the TNF-α stimulus induced a significant upregulation of these targets compared to the control cells in differentiated SH-SY5Y (Figure 4) and in HMC3 cells (Figure 5). Instead, when cells were treated with KJ3 or KJ9 protein expression of p-NF-kB and p-IkB-α was markedly reduced compared to stimulated cells (Figure 4 and Figure 5). 

**Figure 6:**
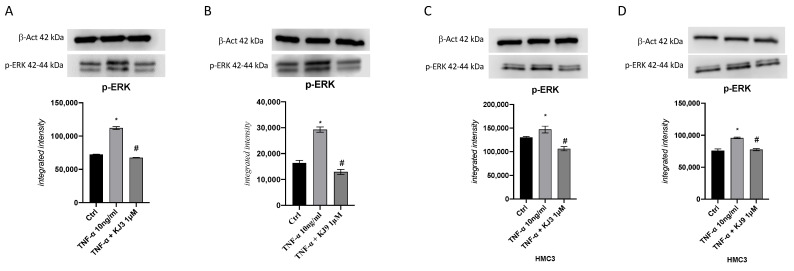
The graphs represent p-ERK protein level assessed by Western blot analysis in differentiated SH-SY5Y (**A**,**B**) and HMC3 (**C**,**D**) cells treated with KJ3 (1 μM) or KJ9 (1 μM) for 24 h following TNF-α (10 ng/mL) challenge. The data are expressed as means ± SDs. * *p* < 0.05 vs. CTRL; # *p* < 0.05 vs. TNF-α.

Neuroinflammation induced with TNF-α was demonstrated also by the significant increase in p-ERK, involved in the inflammatory pathway in neuron and microglial cells. In addition, in this context, KJ3 and KJ9 markedly reduced p-ERK protein levels (Figure 6).

**Figure 7:**
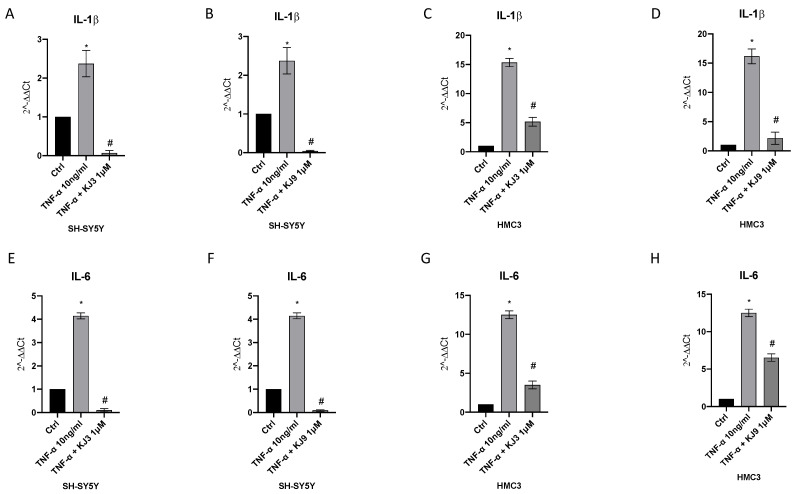
mRNA level of IL-1β and IL-6 evaluated by qPCR in analysis in differentiated SH-SY5Y (**A**,**B**,**E**,**F**) and HMC3 (**C**,**D**,**G**,**H**) cells treated with KJ3 (1 μM) or KJ9 (1 μM) for 24 h following TNF-α (10 ng/mL) challenge. The data are expressed as means ± SDs. * *p* < 0.05 vs. CTRL; # *p* < 0.05 vs. TNF-α.

TNF-α challenge and the consequent NF-kB activation also produced a marked increase in IL-1β and IL-6 RNA levels compared with untreated cells in both cell lines; on the contrary, the level of these pro-inflammatory cytokines was significantly reduced by the amide derivatives compared to the stimulated cells with TNF-α in differentiated SH-SY5Y and HMC3 cells (Figure 7). 

### 2.4. KJ3 and KJ9 Amide Derivatives Modulate the Ubiquitin Levels in Differentiated SH-SY5Y and HMC3 Stimulated with TNF-α

**Figure 8:**
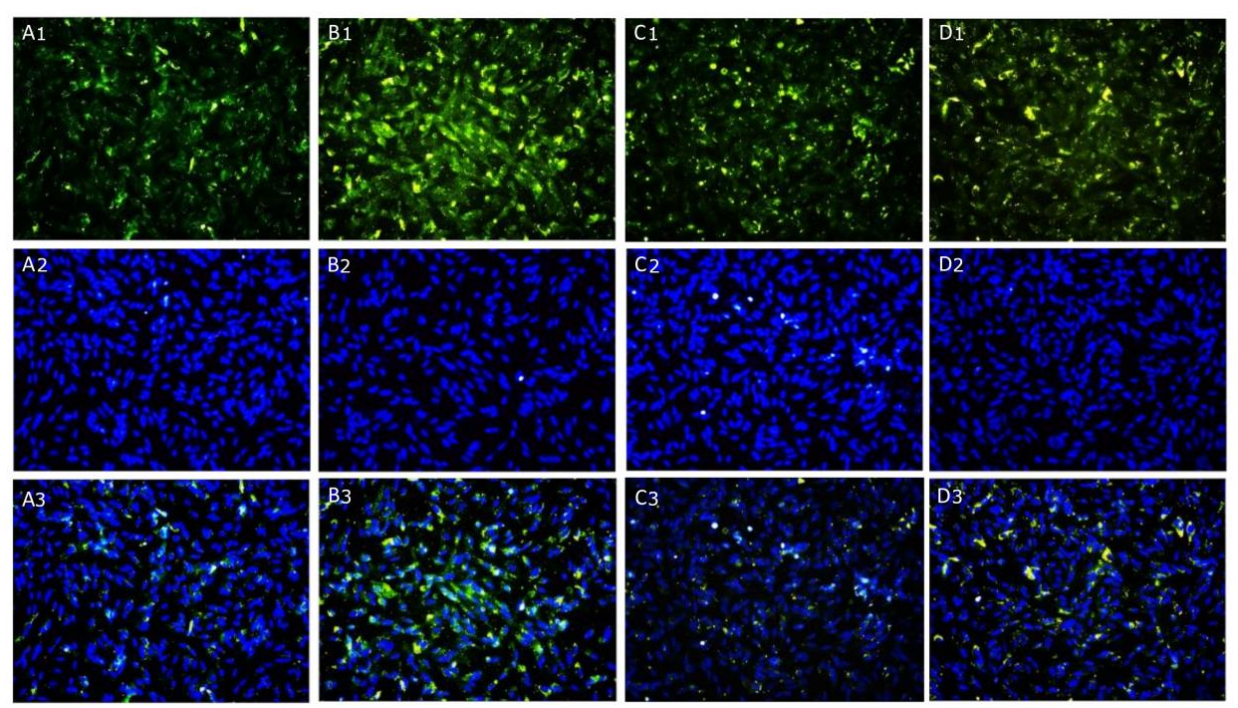
Compound panel of immunofluorescence reactions using an anti-ubiquitin antibody (green fluorescence) in differentiated SH-SY5Y cells (**A1**–**A3**) treated with KJ3 (**C1**–**C3**) or KJ9 (**D1**–**D3**) for 24 h following TNF-α (**B1**–**B3**) stimulation.

**Figure 9:**
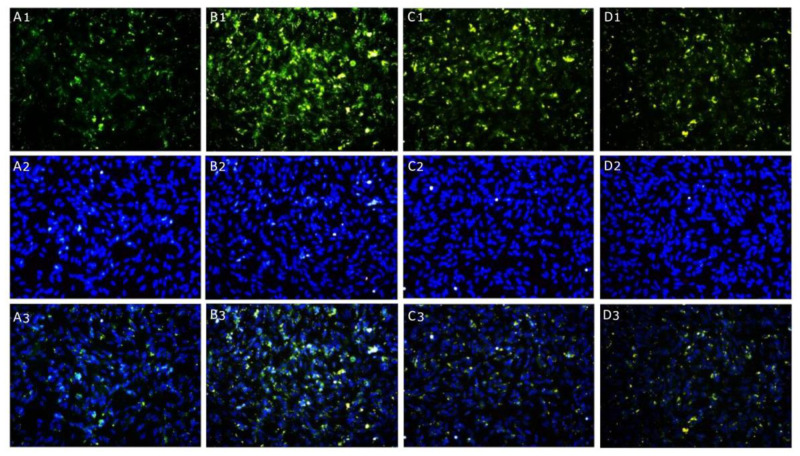
Compound panel of immunofluorescence reactions using an anti-ubiquitin antibody (green fluorescence) in HMC3 cell line (**A1**–**A3**) treated with KJ3 (**C1**–**C3**) or KJ9 (**D1**–**D3**) for 24 h following TNF-α (**B1**–**B3**) stimulation.

The protein expression of ubiquitin, involved in proteasomal degradation of protein, was evaluated by immunofluorescence staining: no fluorescent pattern was detected in control cells of differentiated SH-SY5Y (Figure 8A1–A3) and HMC3 cells (Figure 9A1–A3); instead, the cytoplasmic localization of ubiquitin was observed in TNF-α challenged cells (Figure 8B1–B3 and Figure 9B1–B3) in both cell lines. The treatment with KJ3 or KJ9 caused a strong reduction in the ubiquitin fluorescence pattern compared to the stimulated cells in both neuronal (Figure 8C1–C3,D1–D3) and microglial cell lines (Figure 9C1–C3,D1–D3).

### 2.5. KJ3 and KJ9 Show Anti-Oxidant Properties

**Figure 10:**
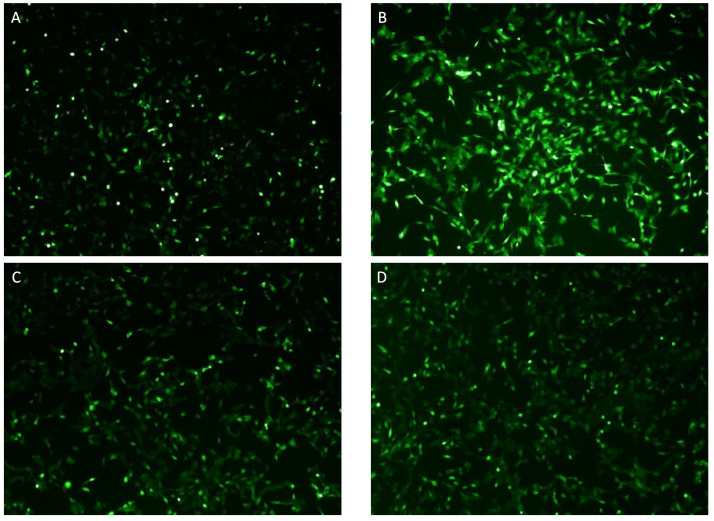
Intracellular ROS accumulation evaluated by a CM-H2DCFDA fluorescent probe in differentiated SH-SY5Y cells (**A**) stimulated with TNF-α (**B**) and then treated with KJ3 (**C**) or KJ9 (**D**) for 24 h. Panel E shows the number of fluorescent cells. All images were captured at 10× magnification.

**Figure 11:**
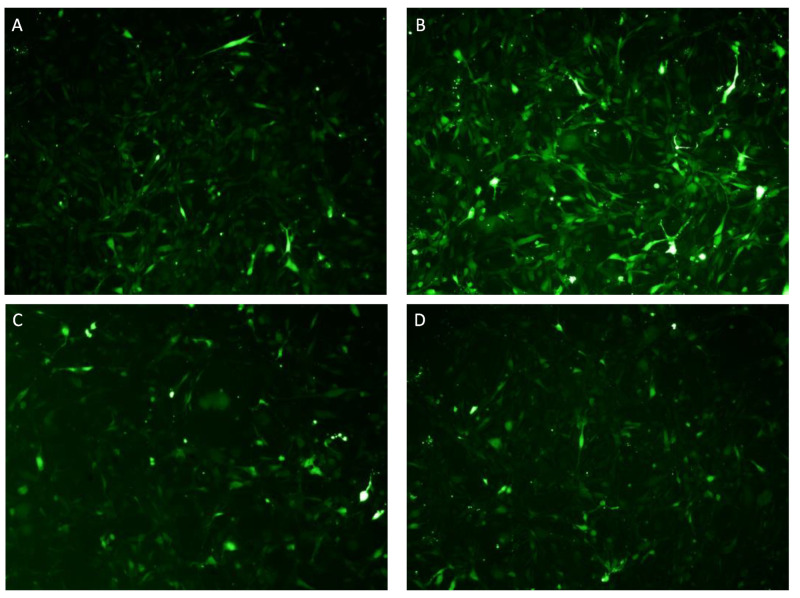
Intracellular ROS accumulation was evaluated by a CM-H2DCFDA fluorescent probe in HMC3 cells (**A**) stimulated with TNF-α (**B**) and then treated with KJ3 (**C**) or KJ9 (**D**) for 24 h. All images were captured at 10× magnification.

Intracellular ROS accumulation was significantly increased when cells were stimulated with 10 ng/mL TNF-α compared to untreated cells in differentiated SH-SY5Y (Figure 10B) and HMC3 cells (Figure 11B). KJ3 (Figure 10C and Figure 11C) and KJ9 (Figure 10D and Figure 11D) markedly decreased ROS levels compared to cells stimulated with TNF-α in both cell lines.

## 3. Discussion

Neuroinflammation is a complex inflammation-like process that plays a key role in the central nervous system (CNS) and it is used by cells of CNS to eradicate pathogens halting the infection, and remove cell debris and misfolded proteins. Overactivated neuroinflammation causes the activation of neuronal cells, such as microglia cells, neurons, and astrocytes, and it is mediated by the release of pro-inflammatory cytokines, such as IL-1β, IL-6, and TNFα, chemokines, reactive oxygen species (ROS), and secondary messengers [16]. The activation of CNS cells during neuroinflammation is caused by several inflammatory stimuli which promote the induction of numerous pathways such as NF-κB and ERK1/2 signaling [17]. In particular, the release of pro-inflammatory cytokines stimulates the MEK/ERK signaling pathway inducing an overproduction and release of TNFα causing the activation of microglia cells [18]. Moreover, Mir et al. demonstrated that IFN-γ and TNF-α play a key role in the induction of oxidative stress, excitotoxicity, and motoneuron death in rat spinal cord embryonic explants [19]. 

The constitutive ubiquitin-proteasome system (UPS) regulates protein homeostasis and is involved in several key processes, such as cell proliferation, cell death, inflammation, and modulation of stressful conditions [20]. High concentrations of pro-inflammatory cytokines (IFN-γ or TNF-α) or oxidative stress caused the development of immunoproteasome (iP) complex in which the active constitutive proteasome subunits, β1c, β2c, and β5c were replaced by β1i, β2i, and β5i. Several pieces of evidence demonstrated that the iP is involved in different inflammatory disorders and regulates ERK1/1, the NF-κB signaling pathway, and pro-inflammatory cytokine production [21,22,23,24,25]. 

Therefore, the use of immunoproteasome inhibitors, able to regulate the activity and the expression of iP, could represent a powerful approach for the control of many cell functions and for the management of several inflammatory diseases.

In this work, we evaluated the properties of two new amide derivatives, KJ3 and KJ9, which act as inhibitors of immunoproteasome, in an in vitro model of neuroinflammation provoked by TNF-α stimulation. In accordance with previous papers and also in our experimental model, TNF-α is able to stimulate neuroinflammation and excitotoxicity in neuronal and microglial cells [21,22,23,24]; indeed, ROS levels and the expression of the inflammatory targets were significantly increased. 

KJ9 was proven to inhibit the chymotrypsin-like activities of β5c and β5i, whereas KJ3 inhibited only the β5i of the immunoproteasome. These data are very important, because it was demonstrated that immunosubunit β5i can control cytokine production through the NF-kB signaling pathway [26,27]. In accordance with this hypothesis, we tested the possible anti-inflammatory effects of these two new amides analogs in neuronal and microglia cells stimulated with TNF-α. Our results demonstrated that KJ3 and KJ9 were able to reduce the expression of p-ERK, IKBα, and NF-kB; moreover, these compounds also caused a significant decrease in IL-1β and IL-6 protein levels, downstream genes of NF-kB, suggesting that KJ3 and KJ9 can reduce neuroinflammation through the modulation of the NF-kB signaling pathway. Thus, these data are in agreement with previous works that demonstrated that other immunoproteasome inhibitors, such as ONX-0914, a peptide epoxyketone-based irreversible β5i-selective immunoproteasome inhibitor, and a dual covalent β5i/β2i selective inhibitor, KZR-616, induce an anti-inflammatory response in an in vivo model of rheumatoid arthritis and inhibit the production of inflammatory cytokines, respectively [28,29,30,31]. 

Several studies demonstrated that diseases characterized by an increase in TNF-α, such as inflammatory disorders, show an increased ubiquitin expression [32,33,34,35]; in particular, TNF-α stimulates the ubiquitin-activating enzyme (E1 protein) that stimulates ubiquitin expression. Activated ubiquitin was transferred to a ubiquitin carrier protein (E2), which interacts with a ubiquitin ligase (E3) and transfers the ubiquitin to the protein substrate [36]. In accordance with these works, in our experimental protocol, TNF-α produced a marked increase in the ubiquitin protein levels in both used cell lines. Moreover, our results showed that in KJ3 and KJ9 treated cells the expression of ubiquitin protein was reduced compared to TNF-α challenged cells; these results are in agreement with Basler et al. that suggested that the ubiquitin did not accumulate in LMP7-deficient cells, probably because constitutive proteasome cope with the immunoproteasome to degrade ubiquitin proteins [37]. Furthermore, it was demonstrated that β5 subunit inhibition does not influence protein degradation and proteotoxicity in multiple myeloma cells [38] whereas Kisselev and Oberdorf suggested that only the inhibition of both β5 and β1 or β2 affects protein degradation and proteotoxic stress [39,40]. To test the involvement of β5i in the degradation of cytosolic and mitochondrial-oxidized protein we evaluated the accumulation of intracellular ROS in differentiated SH-SY5Y and HMC3 cell lines. In agreement with the results obtained for the ubiquitin expression, KJ3 and KJ9 treated cells did not show an accumulation of intracellular ROS confirming the hypothesis that β5 subunit is not involved in proteotoxic stress.

In conclusion, the present study provides evidence that both KJ3 and KJ9, non-covalent β5i inhibitors, own anti-inflammatory activities, since they reduce pro-inflammatory cytokines and regulate the NF-kB signaling pathway, without affecting the ability of immunoproteasome to degrade ubiquitin protein and control proteotoxicity. Moreover, unlike other immunoproteasome inhibitors, these new amide derivatives do not show the drawbacks and side effects due to the non-covalent inhibition.

Considering these results, immunoproteasome inhibitors could represent promising candidates for preclinical and clinical investigations for several human diseases, such as neurodegenerative disease, autoimmune diseases, and inflammatory diseases.

## 4. Material and Methods

### 4.1. In Vitro 20S Immunoproteasome/Proteasome Inhibition Assays

Human 20S proteasome (isolated from human erythrocytes) and human 20S immunoproteasome (isolated from human spleen) were purchased from Enzo Life Science Supplier (Executive Blvd, Farmingdale, NY, USA). The two distinct proteolytic activities of proteasome and immunoproteasome were measured by monitoring the hydrolysis of the peptidyl 7-amino-4-methyl-coumarin substrate Suc-Leu-Leu-Val-Tyr-AMC (Bachem, Bubendorf, Switzerland) for both β5c and β5i activities. 

Inhibitor solutions were prepared from stocks in DMSO. Each independent assay was performed in duplicate in 96-well plates with a total volume of 200 µL. In more detail, we used 5, 10, 20, 40, 60, 80, and 100 µM for both KJ3 and KJ9. An equivalent amount of DMSO was used as a negative control, while MG-132 was used as a positive control. 

IC50 values ± SD and were calculated by fitting the progress curves to the 4-parameter IC50 equation by Grafit software (Version 5.0; Erithacus Software Limited, East Grinstead, West Sussex, UK), with y [∆F/min] as the substrate hydrolysis rate, ymax as the maximum value of the dose−response curve, measured at an inhibitor concentration of [I] = 0 µM, ymin as the minimum value, obtained at high inhibitor concentrations, and s as the Hill coefficient. 

Fluorescence of the product AMC of the substrate hydrolyses was measured using an Infinite 200 PRO microplate reader (Tecan, Männedorf, Switzerland) at 30 °C with a 380 nm excitation filter and a 460 nm emission filter.

### 4.2. Assaying the Chymotrypsin-Like Activity of β5i Subunit of 20S Immunoproteasome and β5c Subunit of 20S Proteasome

Human 20S immunoproteasome or human 20S proteasome was incubated at 30 °C at a final concentration of 0.004 mg/mL with test compound present at variable concentrations. The reaction buffer consisted of 50 mMTris HCl, pH 7.4, 10 mM NaCl, 25 mM KCl, 1 mM MgCl_2_, 0.03% SDS, and 5% DMSO. Product release from substrate hydrolysis (50 μM) was monitored continuously over a period of 10 min.

### 4.3. Cell Cultures 

Human neuroblastoma cell line (SH-SY5Y) and human microglial cells (HMC3) were obtained by ATCC (ATCC Manassas, Manassas, VA, USA). Differentiated SH-SY5Y were cultured with formulated Eagle’s minimum essential medium and F12 medium (EMEM/F12) in a 1:1 mixture; HMC3 cells were cultured with EMEM (Sigma-Aldrich, St. Louis, MO, USA). Then 1% of penicillin/streptomycin antibiotic (Sigma-Aldrich, St. Louis, MO, USA) and 10% fetal bovine serum (FBS) (ATCC Manassas, Manassas, VA, USA) were added to both culture media and cells were incubated at 37 °C with a percentage of 5% CO_2_. The culture’s medium was substituted every two days.

### 4.4. Cell Treatments

SH-SY5Y neuroblast-like cells were plated in a 6-well plate at a density of 1 × 10^5^ cells/well and incubated at 37 °C with a percentage of 5% CO_2_ overnight. The day after, cells were differentiated in neurons-like cells. It has been demonstrated that the brain-derived neurotrophic factor (BDNF) can further increase the established effects of retinoic acid (RA) on neuronal differentiation, making it the most widely acknowledged procedure for differentiation. In particular, 10 µM of retinoic acid (RA) was added to each well for five days changing the medium every two days. On day 6, RA was removed, cells were washed three times with sterile phosphate-buffered saline (PBS), and brain-derived neurotrophic factor (BDNF) at the concentration of 50 ng/mL was added for additional five days.

Differentiated SH-SY5Y cells and HMC3 cells were challenged with tumor necrosis factor (TNF-α) at the dose of 10 ng/mL to induce an inflammatory phenotype for 24 h. TNF-α dose was titrated against the effects on IL1-β expression (Figure S1).

The day after, cells were treated with KJ3 (1 µM) and KJ 9 (1 µM) for an additional 24 h to evaluate their anti-inflammatory effects. At the end of the treatment period, differentiated SH-SY5Y cells and HMC3 cells were collected and used for further molecular analyses.

### 4.5. MTT Assay

Cell viability was evaluated by the MTT assay (3-(4,5-Dimethylthiazol-2-yl)-2,5-Diphenyltetrazolium Bromide) to investigate TNF-α, KJ3 and KJ9 cytotoxicity. In detail, both cell lines were seeded at a density of 1 × 10^5^ cells/well in a 96-well plate; upon reaching confluence, cells were treated with TNF-α (5, 10, 20, and 30 ng/mL), and the amide derivates, KJ3 and KJ9, at different doses (1, 10, 20, 40, 80, and 100 μM) for 24 h. Then 5 mg/mL of tetrazolium dye MTT 3-(4,5-dimethylthiazol-2-yl)-2,5-diphenyltetrazolium bromide (Sigma Aldrich, St. Louis, MO, USA) was thawed in sterile PBS and 20 μL was added into each well five hours before the end of the treatment. Twenty-four hours after starting treatment, formazan crystals were thawed using 200 µL dimethyl sulfoxide (DMSO). Cytotoxicity was quantified using a VICTOR Multilabel Plate Reader (Perkin Elmer; Waltham, MA, USA) at λ 540 and 620 nm. Data are expressed as the percentage of cell viability compared to control cells.

### 4.6. Intracellular ROS Production

To investigate the antioxidant effects of KJ3 and KJ9, intracellular ROS production was assessed employing an 5-(and-6)-chloromethyl-2′,7′-dichlorodihydrofluorescein diacetate (CM-H2DCFDA) probe in differentiated SH-SY5Y and HMC3 cells stimulated with TNF-α and treated for 24 h and treated with KJ3 or KJ9. Fluorescein diacetate (FDA) is a cell-permeant esterase substrate that can be used as a viability probe able to measure both enzymatic activities, indispensable to activate its fluorescence, and cell-membrane integrity, necessary for the retention of their fluorescent product within the cells. This AM ester is hydrolyzed by intracellular esterase to produce fluorescein. The increase in fluorescence due to the oxidation of this probe can be detected with a fluorescence microscope, using excitation sources and filters appropriate for fluorescein (FITC). At the end of the treatment period, cells were incubated with 5 μL of CM-H2DCFDA probe (Thermo Fisher, Carlsbad, CA, USA) for 1 h at 37 °C with a percentage of 5% CO_2_. After three washing with sterile PBS, cells were observed with a fluorescent microscope and the fluorescent intensity was quantified using ImageJ 1.53e software for Windows (Softonic, Barcelona, Spain) [41].

### 4.7. Real-Time PCR Assay 

At the end of the treatment period, differentiated SH-SY5Y and HMC3 cells were scraped employed TRIzol RNA Isolation Reagents (Thermo Fisher Scientific, Waltham, MA, USA), total RNA was extracted from and quantified by UV-Vis microvolume spectrophotometer (NanoDrop Lite, Thermo Fisher, Waltham, MA, USA). A Superscript IV Master Mix (Invitrogen, Carlsbad, CA, USA) was used to reverse transcribe the total RNA (1 µg) in cDNA. The obtained cDNA (1 μL) was added to the BrightGreen qPCR Master Mix (ABM, Richmond, Canada) to assess the mRNA level of IL-1 β and IL-6. qPCR reaction was monitored by using the QuantStudio 6 Flex Real-Time PCR System (Applied Biosystems, CA, USA), the GAPDH gene was used as the housekeeping gene, and the amplified PCR products were calculated by measuring the calculated cycle thresholds (CT) of target genes and GAPDH mRNA. After normalization, the mean value of the normal control target levels was chosen as the calibrator and the results were expressed according to the 2^−ΔΔCt^ method, as a fold change relative to normal controls [42,43,44,45].

### 4.8. Enzyme-Linked Immunosorbent Assay (ELISA)

Following the 24 h of treatment, cells were collected from each well and NFKB protein level was measured from cell lysate employed enzyme-linked immunosorbent assay (ELISA) kits (Abcam, Cambridge, UK), in accordance with the guidelines given by the producer [45]. In detail, standards, the sample and the antibody cocktail were added into appropriate wells. After incubation, wells were washed with wash buffer PT (350 µL) with a multi-channel pipette and 100 μL of TMB substrate was added to each well. The plate was then incubated for 15 min in the dark on a plate shaker set to 400 rpm. After that, 100 μL of stop as wwere added into each well, the plate was shaken on a plate shaker for 1 min to mixm and the OD was measured at 450 nm using a microplate reader (PerkinElmer, Waltham, MA, USA).

All the samples were assessed in duplicate, and the obtained data were interpolated with the appropriate standard curves. Means of the duplicated samples were used to assess NFKB level and express in pg/mL.

### 4.9. Western Blot 

At the end of the treatment period, differentiated SH-SY5Y and HMC3 cells were scraped using RIPA buffer (25 mM Tris/HCl, pH 7.4; 1.0 mM EGTA; 1.0 mM EDTA) with NP40 (1%), phenyl methylsulfonyl fluoride (PMSF, 0.5%), aprotinin, leupeptin and pepstatin (10 μg/mL each) and centrifuged at 15,000 rpm for 15 min at 4 °C to obtain the supernatant from each sample. The Bio-Rad protein assay kit (BioRad, Hercules, CA, USA) was used to measure the total protein content in each supernatant. Then 30 μg of proteins were loaded and resolved by electrophoresis in a 10% sodium dodecyl sulfate (SDS) polyacrylamide gel and transferred onto a PVDF membranes (Amersham, Little Chalfont, UK) using a specific Transfer Buffer at 100 V for 1 h. Following three washing with TBS 0.1% Tween buffer, membranes were blocked with 5% non-fat dry milk in TBS 0.1% for 1 h at room temperature (RT). After washing, the membranes were incubated with specific primary antibodies for p-ERK, IkB-α, and p-NFkB diluted in TBS-0.1% Tween overnight. The day after, the membranes were washed three times with TBS-0.15% Tween buffer and a secondary goat anti-rabbit antibody (GeneTex, Irvine, CA, USA) conjugated with peroxidases was used for 1h at RT to bind and detect the target proteins. Following three washes with TBS-0.15% Tween buffer, membranes were analyzed by the enhanced chemiluminescence system (LumiGlo reserve; Seracare, Milford, MA, USA) and the protein signal were quantified by a scanning densitometry system (C-DiGit, Li-cor, Lincoln, NE, USA). Results were expressed as relative integrated intensity using β-actin (Cell Signaling, Danvers, MA, USA) as a control for the equal loading of samples [46,47,48,49,50].

### 4.10. Immunofluorescence 

Neuronal and microglial cells were cultured at a density of 2.5 × 10^4^ cell/well in 8-well chamber slides and treated with KJ3 and KJ9 for 24 h following TNF-α stimulation. At the end of the treatment period, cells were fixed with 4% of paraformaldehyde (PFA) in 0.2 M phosphate buffer (pH 7.4) for 10 min at RT; then, cells were rinsed 3 times for 10 min with sterile phosphate-buffered saline (PBS). Cells were preincubated with 0.3% triton X-100 in PBS for 10 min to permeabilize the membranes and with 1% bovine serum albumin (BSA) in PBS, for 1 h at RT, in order to block nonspecific binding sites. Cells were then incubated with a rabbit polyclonal anti-ubiquitin antibody (1:250 dilution) (Abcam, Cambridge, UK) overnight at 4 °C. The day after, cells were washed with PBS and a FITC-conjugated IgG anti-rabbit antibody was added (GeneTex, Irvine, CA, USA) for 1 h at RT in order to detect the primary antibody. Nuclei were stained with DAPI diluted to 1:1000 in PBS (Thermo Fisher Scientific, Carlsbad, CA, USA) for 10 min RT. Finally, cells were washed in PBS, the coverslips were mounted on slides, and cells were monitored using a fluorescent microscope. Digital images were cropped, and figure montages were prepared using Adobe Photoshop 7.0 (Adobe System, Palo Alto, CA, USA) [51].

### 4.11. Statistical Analysis 

All data are expressed as mean ± standard deviation (SD). The testified values are the results of at least three experiments. All assays were performed in duplicate to guarantee reproducibility. The differences between the groups were evaluated by one-way ANOVA with a Tukey’s post-test. A *p*-value less than 0.05 was considered significant. Graphs were prepared using GraphPad Prism Version 8.0 for macOS (GraphPad Software Inc., La Jolla, CA, USA.

## Data Availability

Data will be made available on request to the corresponding author.

## Supplementary Materials

The following supporting information can be downloaded at: https://www.mdpi.com/article/10.3390/ijms241310732/s1.
