# Supplementary figures and images for: Blunting Neuroinflammation by Targeting the Immunoproteasome with Novel Amide Derivatives
Source: Int J Mol Sci. 2023 Jun 27;24(13):10732. doi: 10.3390/ijms241310732 (PMC10342075; doi:10.3390/ijms241310732)

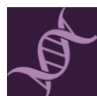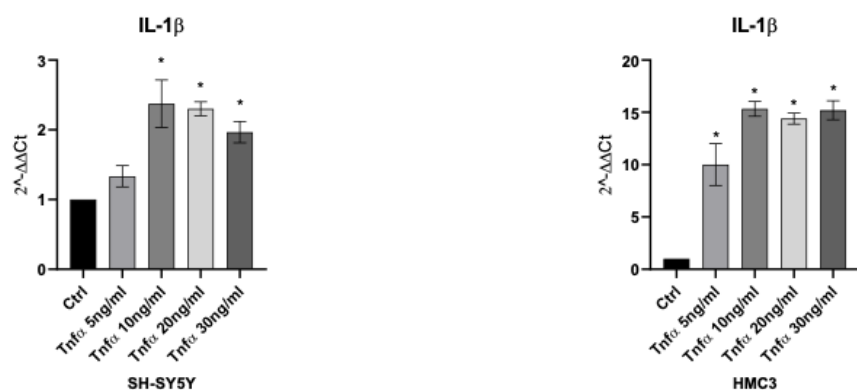

**Figure S1.** TNF- $\alpha$  dose was titrated against the effects on IL1- $\beta$  expression.

Supplement: Supplementary file 1 [file ijms-24-10732-s001.zip › ijms-2451894-supplementary.pdf]
